# The End of the Elimination Strategy: Decisive Factors towards Sustainable Management of COVID-19 in New Zealand

**DOI:** 10.3390/epidemiologia3010011

**Published:** 2022-03-18

**Authors:** Alicia Blair, Mattia de Pasquale, Valentin Gabeff, Mélanie Rufi, Antoine Flahault

**Affiliations:** 1 Global Studies Institute, University of Geneva, 1205 Geneva, Switzerland; alicia.blair@etu.unige.ch (A.B.); mattia.de-pasquale@etu.unige.ch (M.d.P.); melanie.rufi@etu.unige.ch (M.R.); 2 Institute of Global Health, University of Geneva, 1202 Geneva, Switzerland; antoine.flahault@unige.ch

**Keywords:** COVID-19, New Zealand, strategy, elimination, long-term sustainability

## Abstract

New Zealand has long been praised for the effectiveness of its COVID-19 elimination strategy. It resulted in fewer COVID-19-related deaths, better economic recovery, and less stringent policy measures within its borders compared with other OECD countries, which opted for mitigation or suppression. However, since September 2021, the rising number of infections has not been contained anymore by the contact tracing and self-isolation system in place and the government has shifted towards a policy strategy similar to suppression to manage the crisis. In this case study, we analyse the factors that led the government to switch policy and discuss why elimination became unsustainable to manage the COVID-19 epidemic in New Zealand. Results showed that the socioeconomic and political factors, along with the appearance of new variants and a delayed vaccination program, were accountable for the switch in strategy. This switch allows the country to better adapt to the evolving nature of the disease and to address the social and economic repercussions of the first year of measures. Our conclusion does not disregard elimination as an appropriate initial strategy to contain this pandemic in the absence of a vaccine or treatment, but rather suggests that borders cannot remain closed for long periods of time without creating social, economical, and political issues.

## 1. Introduction

To contain the worldwide spread of COVID-19 in the first months of 2020, and in the absence of a vaccine or efficient treatments, governments implemented various non-pharmaceutical interventions (NPIs) such as lockdowns, quarantines, and border restrictions. These decisions were often accompanied by debates on their duration and intensity, explained by competing interests between stopping the pandemic and accounting for people’s social, political and economic needs. New Zealand’s reliance on an “elimination” strategy turned out to be successful in protecting its population during 2020. However, the coronavirus pandemic is still ongoing and governments must now reckon with long-term crisis management, not just with short-term crisis management.

In this study, we explore which factors initiated the elimination strategy in early 2020 and why it was the appropriate answer. We then show how the evolution of some factors, as well as the current COVID-19 epidemiological situation, impacted the functioning of the elimination strategy and made it unsustainable to manage the pandemic in New Zealand in the long term. We specifically pay close attention to the switch of policy strategy announced by the government in October 2021 and discuss what can be learned from this decision for future pandemics.

### Context

New Zealand is an ensemble of islands, lying isolated in the southwest Pacific Ocean with a distance of about 1600 km from Australia [1]. The total population was 5,084,300 in 2020 according to the World Bank database [2], which represents about 19 people per square kilometer. This figure seems deceiving, as about one third of New Zealand’s population lives in Auckland [3]. The 2018 census reveals that the population is made up of six major ethnic groups. The majority of New Zealand’s population is of European descent (70.2%), while the indigenous Māori make up the largest minority (16.5%), followed by Asians (15.1%), Pacific peoples (8.1%), Middle Eastern, Latin American and African people (1.5%), and people identifying with another ethnicity (1.2%) [3].

New Zealand is governed by a prime minister (PM); the current PM has been Jacinda Ardern (Labour Party) since 2017. Two particularities of the New Zealand political system are the following: the use of mixed-member proportional (MMP) elections, that encourage coalition building; and the centralization of the decision making, with the absence of a regional-level government [4].

New Zealand is part of the Organisation for Economic Cooperation and Development (OECD), with its GDP similar to the group average. The national economy relies significantly on tourism, comprising almost 10% of direct and indirect contributions [5]. Moreover, almost 14% of working residents are employed by the tourism industry [5]. Finally, unemployment was at 4% in December 2019, and while it increased up to 5.3% in September 2020, it is back down to 3.4%, as of September 2021 [6].

New Zealand’s healthcare (HC) system, also referred to as the New Zealand health and disability system, has been built on more than 20 items of legislation throughout its history. In particular, the New Zealand Public Health and Disability Act, which came into force in 2000, mainly establishes District Health Boards (DHB), which monitor the healthcare system improvement over time, providing high-quality and accessible healthcare for local communities, and ensures the implementation of national HC policies at the local level [7]. One of the targets of the act is to reduce disparities among the population [8]. In 2019, New Zealand had an average of 3.6 intensive care beds for 1,000,000 inhabitants, while the OECD average was 12 beds, showing that there is a lack of hospital beds in the country [9]. Nevertheless, in terms of physicians and nurses, New Zealand performs better than the other OECD countries with its 3.59 (2.9 for the OECD) physicians [10] and 12.4 (9.6 for the OECD) nurses and midwives per 1000 people in 2018 [11]. These numbers confirm the strengths, as well as the margins for improvement, of the healthcare system in New Zealand.

The first case of COVID-19 on the New Zealand territory was reported on 28 February 2020 [12]. Until August 2021, the choice of an elimination strategy to contain the COVID-19 pandemic was proven to be particularly effective, with less than 600 cases and 5 deaths per million inhabitants [13]. The incidence of the disease was by far the lowest among the OECD countries and the choice of such a strategy greatly accounts for this difference [14]. Aside from two small and quickly contained outbreaks in August 2020 and February 2021 in Auckland, the country experienced no internal circulation of the virus until the summer 2021 [13].

**Figure 1:**
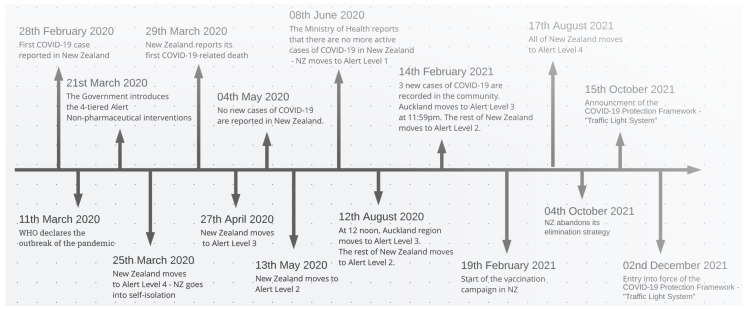
Timeline with dates of key events and policy decisions taken by the New Zealand government. On March 25, New Zealand moved to Alert Level 4 due to a high incidence of daily SARS-CoV-2 cases. With the growing governmental stringency being implemented, the Alert Level returned to 1 within June 2021. Both in August 2021 and February 2021, Auckland moved to Alert Level 3, while the rest of the country reached Alert Level 2, with a higher stringency in accordance with Figure 2. In August 2021, the whole country moved to Alert Level 4, associated with a sharp rise of the incidence and stringency index.

Nonetheless, increased transmissibility of the virus resulted in a rebound of the number of cases in August 2021 and the alert system in place failed to break the chains of transmission of the disease. This outbreak is still ongoing and the country is now experiencing its highest number of daily cases since the beginning of the pandemic. As of the end of October 2021, New Zealand had accumulated a total of less than 1300 cases per million inhabitants and 35 deaths [13]. To adapt to the situation, the New Zealand government revised its strategy towards what we consider a suppression model and replaced its alert system to a more flexible three-level protection framework on 22 October 2021 [15]. Officially, the new strategy is called “Minimisation and protection” [16]. The vaccination campaign has followed along since February 2021 and more than 60% of the population had received a complete immunisation by the end of October [13]. Despite a slow vaccination rollout during spring, it quickly caught up during the summer and fall. Figure 1 summarises the succession of policy decisions affecting the epidemiological situation of the country over time.

## 2. Materials and Methods

The materials used for this study are based on the research of relevant articles published in peer-reviewed journals, articles published in lay press, and the information available through the New Zealand government’s website. For official statistics, the study relies on information drawn from the New Zealand Stats website as well as the Our World in Data website [13]. For this paper, real-time data was taken into account until the end of October 2021. As for the selection criteria, we only considered materials in English, and we looked at academic papers dealing with the COVID-19 pandemic in New Zealand published between March 2020 and the end of October 2021. We excluded editorials and letters to the editor. To search for academic literature, Google Scholar and PubMed search engines were employed with the initial following keywords: “New Zealand”, “COVID-19”, “Strategy”, and “Evolution”. Four of the authors worked on the literature review, while focusing on the specific topics they contributed to in this paper. In addition to scientific input drawn from peer-reviewed material, we benefited from press releases and comments from lay press articles which provided additional insight particularly useful for public policy analyses.

## 3. Results

### 3.1. The Initiation of the Elimination Strategy

As soon as March 2020, New Zealand decided to implement an “elimination” strategy [16], which is defined as: “Reduction to zero of the incidence of a specified disease in a defined geographical area as a result of deliberate efforts, continued intervention measures are required.” [15]. There are multiple factors, specific to New Zealand, that influenced the government’s choice.

First of all, New Zealand is an island—implying the absence of borders that could be easily crossed—it is relatively remote, and not densely populated. Because of this remoteness, the spread of COVID-19 in the country was delayed compared with the rest of the world, which allowed New Zealand time to observe and prepare; it also allowed them to close off borders more successfully [18]. This decision was also influenced by the characteristics of the disease. Evidence from China and following studies showed that the original strains of the severe acute respiratory syndrome coronavirus 2 (SARS-CoV-2) was overall more dangerous than influenza. Although SARS-CoV-2 had a milder severity, influenza had a lower reproductive rate (i.e., 1.5 for influenza viruses vs. 2.4 for the original strain of SARS-CoV-2), did not exhibit overdispersion of the R0, and had a shorter incubation period (1–2 days vs. 5 days on average) [19,20]. The 2017 Aotearoa New Zealand preparation plan for pandemics was based on a mitigation strategy which is efficient against influenza-like disease [21]; however, this plan was not suited for COVID-19. A “go hard, go early” elimination strategy was chosen in March 2020 to temporarily contain the pandemic [22]. This decision also corresponds to the state of the national healthcare system. The low intensive care bed capacity, representative of a general underfunding of public health, was a cause for concern, as the healthcare system did not have the resources needed to handle a pandemic of this scale [9,18]. Taken together, these elements justified the implementation of the elimination strategy aiming to reduce the incidence of the disease to zero in the territory before acquiring enough pharmaceutical and individual protections to end the epidemic in New Zealand.

### 3.2. The Implementation of the Elimination Strategy

According to Baker et al, several key components are necessary to make an elimination strategy efficient [19,23]. First, adequate communication between decision makers, scientists, and the population is essential to make valuable decisions that are widely accepted by the public opinion. Second, careful control of disease evolution is performed through strict border closure. Only New Zealand residents and citizens are allowed to enter the country with very few exemptions possible, such as air crews. Fast determination of chain of transmissions through contact tracing and self-isolation are used to follow and contain the propagation of the disease within the territory. Outbreaks will inevitably occur but should be quickly controlled by implementing geographically targeted measures. Third, investments for an operational healthcare system, as well as population immunisation and NPIs are needed to reduce transmission of the virus and to quickly detect new cases through testing. Additionally, measures should be taken to preserve economic growth and social equity.

All these points were successfully addressed by the New Zealand health authorities. Since 14 March 2020, a fourteen-day self-isolation period is required for anyone entering the territory except from Pacific regions. This was quickly followed by a ban on gatherings of more than 100 people and a complete closure of borders to any non-resident or citizen. Few days later, a strict border control was put into place and entrance has only been allowed for New Zealand citizens and permanent residents ever since [12]. The government declared a state of emergency to quickly implement health measures on 25 March 2020. A four-level alert system was defined to accurately and quickly reduce incidence. This system led to the announcement of several local or national lockdowns to control outbreaks when contact tracing and self-isolation were not sufficient to stamp out chains of transmissions.

In 2020, New Zealand received praise from the WHO for its successful elimination of the virus [24]. Before the spread of the Delta variant in late summer 2021, the country boasted remarkable figures: less than 3000 total cases as of 1 August 2021, and 26 deaths [13]. Moreover, except for border restrictions, the population of New Zealand was able to live in relative freedom, with month-long periods of time free from any restriction [18]. Additionally, the elimination strategy was associated with a better economic recovery than countries opting for different strategies and required less stringent measures in the long term than suppression or mitigation strategies [25].

The first element that facilitated the success of elimination is the country’s government structure and political culture. As previously mentioned, the New Zealand government is centralised, and there are no regional governments. Following this pre-existing governmental organisation, the COVID-19 response had a top–down dynamic, which allowed for faster and more decisive decision making from the central government, and granted more authority to the PM [4]. The government’s culture of coalition building also probably facilitated the agreement between parties. In addition, the decision-making process was supported by scientists, including the Ministry of Health’s COVID-19 Technical Advisory Group [18]. The implementation of measures was facilitated by a generally compliant population [18]. Indeed, it seems that the population has a high level of trust in their leaders: a poll in July 2020 showed that 83% of respondents agreed that the government was “generally trustworthy” [26]. Moreover, 78% find that the management of the pandemic has increased their level of trust [26]. Although these numbers reflect the trust after the start of the elimination strategy, we suggest that trust was already decently high in March 2020. It is likely that trust increased after the implementation of an effective strategy, and that similarly this implementation was first possible because of public trust [26].

The level of compliance probably benefited from the Cabinet’s communication, which was clear, transparent, and meant to provide one reliable source of information that people could trust [18]. The evidence and reasoning behind decisions were explained, betting on people’s capacity for rational understanding [18]. The messaging also focused on solidarity and fairness by referring to New Zealand as the “team of 5 million”, and reduced politicisation [18].

At the start of the pandemic, the country’s backward contact tracing capacities were insufficient. New Zealand’s twelve regional Public Health Units are usually responsible for contact tracing; however, the large number of cases coming in from abroad in early March 2020, before strict border closure, was beyond their abilities [27]. The creation of a National Close Contact Service in March, the development of new technological solutions, and a wider staff dedicated to tracing were meant to increase capacities [27]. Millions were invested in public health, including contact tracing [28]. Efficient and quick contact tracing is absolutely necessary to manage infectious diseases [29], especially when lockdowns are lifted [27]. It seems that these investments, combined with lower number of cases after the first wave, resulted in a performant contact tracing system, as it successfully identified the members of clusters [29]. It should be noted that the efficiency of contact tracing was most likely not linked to the development of a tracing smartphone application [30,31]. The existence of affordable healthcare also facilitated the participation of the population in the elimination strategy, as affordable care allows for better self-reporting [4].

### 3.3. Evolution of the Context and Adaptation of the Strategy

Several factors have evolved between the beginning of the pandemic and October 2021, making the elimination strategy less sustainable in the long term. This evolution has caused changes in the response strategy, as we discuss now. The new strategy explained here was decided in October 2021 at the latest; the evolution of the pandemic might cause these plans to change.

#### 3.3.1. Epidemiological Evolution

Delta variants (B.1.617.2 and AY lineages) of SARS-CoV-2 emerged in December 2020 and are considered variants of concern (VOC) [32]. Those variants have indeed demonstrated increased transmissibility, stronger resistance to antibodies, and increased severity, posing a threat to the management of the pandemic [33]. Delta variant structure makes it also more likely to reduce the effectiveness of previously developed vaccines, reducing the efficacy of the vaccination campaign [33]. The Delta variant has been associated with several outbreaks in New Zealand since August 2021, and represented 100% of all the strains of SARS-CoV-2 in positively diagnosed patients as of October 2021 [13]. The increased transmissibility of the virus due to the appearance of the Delta variant and the low proportion of vaccinated people in August 2021 resulted in a failure of the elimination strategy to contain the epidemic. Indeed, the pace of new cases detection, contact tracing and self-isolation were not sufficient to break chains of transmission at their early stage. Only strong lockdown measures are able to stamp out transmissions when critical rates are attained, which is also the option chosen by countries following a mitigation strategy.

**Figure 2:**
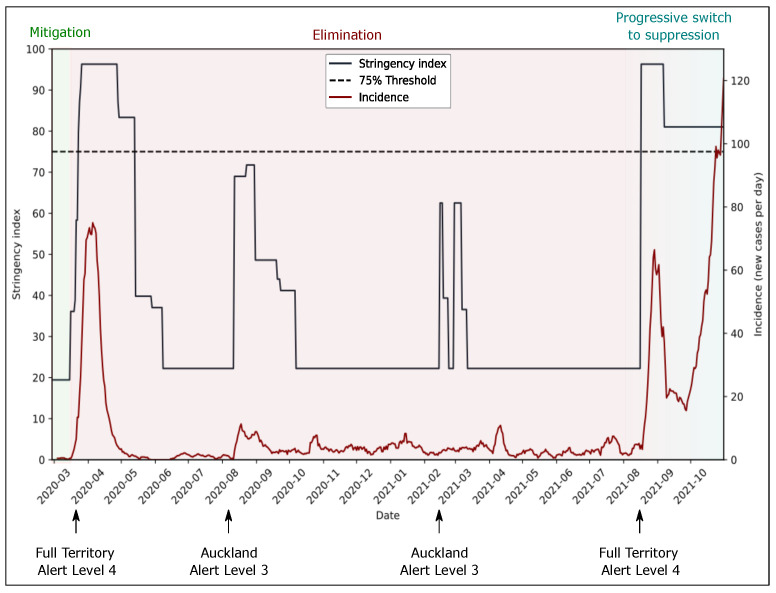
Evolution of governmental stringency index and incidence of daily SARS-CoV-2 cases over time in New Zealand along with adopted policy strategies. From the definition elaborated by Hale et al., the stringency index is a composite measure of 9 response metrics capturing closing and containment measures to represent the restrictiveness of policy measures on a scale from 0 to 100 [17]. The incidence corresponds to the number of new SARS-CoV-2 cases per day corrected with a 7-day rolling average [13]. An arbitrary threshold of 75 was set for the stringency index to highlight periods that had strong governmental measures associated with high incidence. Incidence was high from March to mid-April 2021 and started rising again in September 2021. The elimination strategy was proven to be effective in lowering the incidence with little restriction during this period of time.

Figure 2 shows the evolution of governmental stringency index along with incidence over time between March 2020 and November 2021 in New Zealand. We can see that increased stringency successfully reduced incidence in March 2020, August 2020, and February 2020, but did not contain the latest increase in cases. Decreased stringency in September and August 2021 combined with a high incidence shows the switch to more relaxed policies. In October 2021 it was formally announced that the measures no longer aimed for zero cases and that the four-level alert system would change into a three-level framework [15].

**Figure 3:**
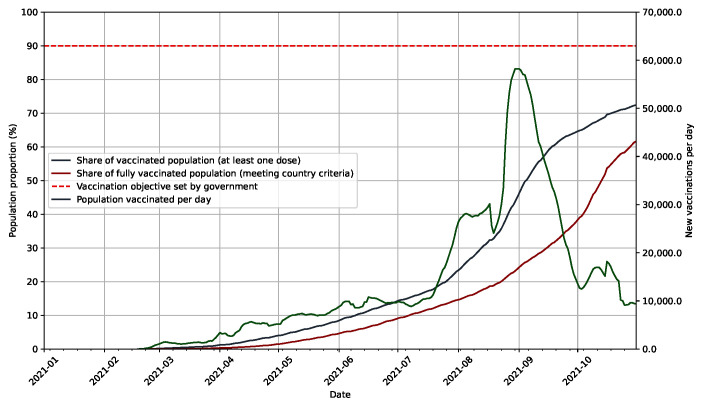
Evolution of vaccinated and fully vaccinated population share over time along with governmental objectives in New Zealand. Since the beginning of the vaccination campaign in February 2021, more than 70% of the population have received one dose of vaccine and more than 60% two doses with a lagging period of about a month and a half. The peak in the number of administered doses early September 2021 corresponds to the opening of vaccination to all adults above sixteen years of age. This number has decreased since and appears steady at around 10,000 vaccinations per day. The speed at which the gap between current vaccination proportion and governmental objective has been decreasing since mid-September 2021. Population vaccinated per day is smoothed with a rolling average window of 7 days.

The vaccination program was initiated by the government in February 2021. Because only older age groups could be vaccinated as of August 2021 [34], only 15% of the population had received at least two doses of COVID-19 vaccines in New Zealand, as shown in Figure 3. This was quickly compensated in September 2021 by the opening of the vaccination to all adults above sixteen years of age, resulting in a peak of administration of new doses. Yet, vaccination rate has been decreasing ever since and reaching the 90% objective set by the government for population immunisation to really be able to reduce transmissions has become more difficult. The goal to immunise 90% of the population is thought to be reached during the first quarter of 2022 [22]. Vaccination programs were attractive to people who were still hesitating, but did not convince those who initially opposed vaccination [35]. The Māori population is slightly less likely to get vaccinated than Asian and other populations [35]. Vaccination was also thought to not be high enough at the time to prevent those outbreaks from spreading and reaching particularly marginalised populations who are known to be more reticent to vaccination [35].

#### 3.3.2. Social Inequalities

Even before the pandemic started, social and health inequities between European New Zealanders and Māori and Pacific peoples have existed within New Zealand concerning for example communicable and non-communicable diseases, access to health, or social determinants of health [36]. Multiple health inequities between Māori and non-Māori have been identified, such as inequity in access to health services or inequity in quality of said services [37].

When the first cases arrived in New Zealand, Māori considered the elimination response to be inadequate since it did not take into account their own situation and it would have a disproportionate impact on the Māori community [38]. For example, the Ministry of Health included people over the age 70 and those with respiratory issues in the group of the “most vulnerable to COVID-19“, but Māori healthcare professionals showed concern that this group did not include Māori at risk population aged 50–60+ years [38]. Although the non-Māori population is structurally older, it also has better access to healthcare than the Māori and other Pacific peoples, which are younger in their age structure but have a higher prevalence of comorbid conditions, which could lead to inequities in COVID-19 fatalities [39]. As long as the overall number of COVID-19 cases remains low, there is a chance that factors such as experience of multi-morbidity or unmet healthcare needs in indigenous populations are less important in the case of COVID-19 [39].

There is also a risk of inequitable vaccine distribution. Individuals should be prioritised based on medical and social vulnerabilities, including the geographical accessibility of healthcare services [40]. As early as May 2021, the Minister of Health acknowledged the importance of ensuring Māori have access to tools and information about the COVID-19 vaccine. Additionally, funding to train Māori health personnel to administer the vaccine and a website with vaccine centres [41] were put into place [42]. More funding from the Ministry of Health also provides financial support for Māori health providers responding to COVID-19 and for Māori communities [43]. As of mid-October, only about 63% of Māori had their first shot compared with 84% of Pakeha (European New Zealanders). An important factor for these vaccination rates plays the geographical distribution of vaccine centres. Only few are set up in rural Māori communities, resulting in a lack of access to vaccines. The situation is exacerbated by high levels of government distrust as well as socioeconomic constraint [44]. A new fund was set up to accelerate Māori vaccinations, where Māori, Iwi (tribes), community organisations, and health providers are funded to prepare for the new protection framework [45].

#### 3.3.3. Pressure at the Borders

Possibly the main feature of the elimination strategy has been the tight closing of New Zealand’s borders. It has been the key to elimination: infected travellers were responsible for introducing the virus in the country, and subsequent border closure and movement restrictions while community transmission was still low [29]. New Zealand has been requiring anyone entering the country to quarantine in an MIQ (Managed Isolation and Quarantine) facility for 14 days, although the number of available rooms is limited. Once elimination had succeeded, travel restrictions allowed New Zealanders to return to a quasi-normal life inside their country. Surveys showed the anxiety at the idea of opening up back to the world, some even describing the border as the country’s Achilles heel [46]. The rest of the world was considered by some as still too dangerous. Respondents were unwilling to risk losing what they had worked so hard for and would rather wait for the vaccine [46].

The context has changed since these surveys were made in 2020; for one, vaccination is ongoing in New Zealand. As the country is accepting the continuous presence of the virus, the question of the borders is more pressing than ever. In October 2021, the government finally announced new, lighter measures, including a shortened quarantine time and a pilot for at-home isolation [47]. Despite the anxiety of the virus and the role the borders played in elimination, New Zealand is slowly opening its doors.

New Zealand greatly relies on tourism income; however, tourism has been almost impossible during the first 18 months of the pandemic. There have also been issues tied to migration status, where people with temporary visas were not able to enter the country if they were abroad at the time of the border closing [48]. As late as November 2021, there has been reporting of skilled worker shortages, in part caused by MIQ policy [49]. This is especially a concern when it comes to health workers, with medical facilities apparently short on staff and unable to bring nurses and doctors into the country [50]. Beyond economic concerns, the bottleneck effect of the MIQ system also has personal consequences. Newspapers are reporting stories of NZ residents, even citizens, unable to come home. MIQ now operates through an online, random lottery, but with 3700 rooms available and several thousands of people entering every time, some people are stuck where they are [51]. The shorter quarantine and at-home isolation recently announced by the government seem more adequate: as this will free up space more quickly, it should reduce “the friction at [the] border“ [52]. Members of the government and advisers have been expressing their desire to open the borders since at least August 2021, adding that the restrictions were always meant to be temporary and that vaccination will allow for a phased reopening [52]. At the time of this speech, the plan was to wait until early 2022 to lift border restrictions and to maintain an elimination strategy. The evolution of the virus and the acceleration of vaccination have prompted the progressive opening of the borders to start earlier than anticipated. However, the point still stands that the population—whether employees, employers, abroad, or at home—have been enduring the weight of being cut off from the world, and that mounting pressure is certainly participating in the end of New Zealand’s isolation.

### 3.4. Healthcare System Reform

The need for a reform of the healthcare system was first discussed in 2018 by the New Zealand Government. The last review of the current healthcare system was published in mid-2020, conducted by the Health and Disability System Review, revealed that there are various opportunities to build a new system that will perform better for all New Zealanders, especially the ones that have been underserved in the past decades. The main goal of the reform is to decomplexify the system, to improve efficiency, consistency, and equity [53]. The aim is to achieve this reform by 2022–2023, despite the COVID-19 pandemic outbreak [54]. Indeed, the authorities decided to keep going with the reform by ensuring a solid and sustained response, as well as strengthening the workforce when necessary [55]. COVID-19 exacerbated the pressure faced by the current healthcare system; therefore, to prevent the situation from worsening over time, the reform had to be pursued during the pandemic for a quick and urgent relief in the near future. According to the Department of the Prime Minister and Cabinet (DPMC) Health Reform White Paper issued in April 2021, the reform is based on four pillars, making the system simpler and ensuring better, more consistent, and sustainable care for all [55].

## 4. Discussion

In this case study, we identified and analysed key factors explaining why the elimination strategy became an unsustainable strategy to address the COVID-19 pandemic in New Zealand, while it was working remarkably well until August 2021. Specifically, the increased basic reproductive number of the new Delta variant, a late vaccination program, the strengthening of social inequalities, and the prolonged closure of territorial borders were all tightly linked to the decision from the government to switch to a policy strategy similar to suppression. Elimination heavily relies on strict border control which eventually failed to contain the disease due to leak of the virus during quarantines rather than violations of quarantine rules. Infectiousness of the virus has a strong impact on the type of measures that can be implemented, and, as we saw, the uncontrolled leaks only occurred because of the Delta variant. Yet, the increase in infectiousness and virulence of the Delta variant and the delayed vaccination coverage were most likely not solely responsible for the switch to the new strategy. Rather, these elements arose at a later stage of the pandemic, when social inequities had had time to exacerbate and prolonged border closure had impacted the economy and social needs of citizens. Pursuing stringent measures, which had become less effective, was not conducive to the well-being of the population anymore. Nonetheless, the elimination strategy was a suitable choice at the early stage of a pandemic as our analysis also highlights. For the majority of the elimination strategy, New Zealand experienced very little deaths and only three localised outbreaks that were quickly contained [13]. It also allowed for less stringent measures and better economical recovery [18,25]. On a more global perspective, it is also an efficient strategy that does not rely on the hurried development of treatment and vaccines to immunise and protect the population, but rather that aims at directly eliminating the disease in little time using only NPIs. This is of particular relevance as we now observe that effective vaccination levels are difficult to achieve in several countries due to poor acceptability by the population or low effectiveness of some vaccines [56]. Vaccines are also expensive and unequally distributed across nations despite attempts to provide low- and middle-income countries with free vaccines [13,57].

Because strict border closure is not sustainable in the long term, an elimination strategy seems effective at the global scale only if every country concerned implements it. The COVID-19 pandemic showed us that while a few countries opted for early stringent measures to initiate elimination or suppression, others used mitigation followed by suppression to avoid taking stringent measures with only few cases on the territory. This let the virus spread in multiple communities and eventually develop mutations that made it more severe and infectious [33]. As a consequence of migration movements, a mutation of the virus, when more transmissible, will inevitably reach countries who initially opted for an elimination strategy, like it is currently the case in New Zealand with the Delta variant and potential new variants. Overall, measures were more stringent in the countries which waited the longest to suppress the virus, with poorer economic recovery, which were initially the reasons to not undergo suppression [25]. Although elimination works well at the country level, it becomes ineffective to manage the evolution of a pandemic if the disease is spreading and mutating in other regions. International collaboration is also important to precisely study the evolution of the different strains over time and their epidemiological consequences. Finally, greater international cooperation would facilitate population movements across more heavily controlled borders, and define agreements on what are essential travel reasons.

Altogether, close monitoring of social and economic factors, as well as epidemiological characteristics, is essential to adapt pandemic response strategies. Opting for a fixed plan cannot well respond to the endemicity of a disease over time. New Zealand’s initial response was so effective precisely because it heavily relied on scientific facts to make decisions [18]. It thus makes sense to pursue this evidence-based approach as the situation evolves. The case of New Zealand also shows that scientific, clear, centralised, and non-politicised communication with the public is essential to garner approval and compliance [18]. This suggests that, aside from the content of a strategy, the way it is discussed also matters—both style and substance have a role to play.

There are also national systemic characteristics that influence the success of a strategy. This paper has highlighted the role that an efficient HC system plays—underdevelopment or backwardness are a threat to the handling of a sanitary crisis. A well-functioning system requires equitable allocation of resources, accessibility, and clarity; otherwise, a reform is needed, as is the case in New Zealand.The country had to face the pandemic with an outdated HC system and must now reform it while handling the virus [55]. For example, New Zealand had the low number of 3.6 ICU beds per 100,000 people [9]. Consequently, the future of the country’s HC will be marked by the pandemic, as the last HC official review was published in 2020, and that the new HC reform was designed in 2021 [54]. For example, the creation of a public health agency is justified through the failures observed in the existing system during the first year of the pandemic [55]. National healthcare systems should account for non-communicable diseases, but also continue to work on infectious diseases. Especially in the face of global catastrophic biological risks such as pandemics, as a part of an efficient HC system, it is crucial to have an operative pandemic plan that is not too pathogen-specific and that offers different pandemic strategies according to the transmission characteristics [21]. The Global Health Security (GHS) Index created by the Nuclear Threat Initiative and the Johns Hopinks’ Centre for Health security identified several weaknesses in New Zealand, such as an understaffed epidemiology workforce, insufficient commitment to share and report surveillance data, and lack of regular exercises to test the response to an emerging biological risk [21]. An updated pandemic plan, even while the COVID-19 pandemic is still ongoing, could address these weaknesses. Another structural aspect is that of inequalities. In New Zealand, access to HC varies depending on social and economic characteristics, especially between Pakeha and Māori populations [37]. Not only does it have moral consequences, as the response to a pandemic might then disproportionately affect marginalised communities, but it also has a strategic impact. The current rollout of vaccines shows that these communities are more difficult to reach, which is a problem since an overall high vaccination rate is necessary to compensate for the end of the elimination measures [44]. Another issue is that Māori were not eligible right away to get the vaccine even though they are at higher risk [58]. The government has been setting up funds and working closely with Māori, Iwi, and community organisations to accelerate the Māori vaccination rate [43,45].

A limitation of this case study is that we can only assume which factors were really taken into account by decision makers, and how they influenced the decisions, or why they took the decisions they did. We have access to their public statements, but it is impossible to know how the truth might differ from what is said publicly. We presented factors coming from both government communication and scientific literature, and they were selected because of their relevance to the topic, with the assumption that this relevance made it likely that they were considered by government officials. Additionally, we aimed for a broad literature review, which may then not be systematic or exhaustive. Furthermore, we did not analyse the factors influencing the slow vaccination rate in the population as we believe them to be numerous and therefore would go beyond the scope of this paper. This analysis could be part of another paper focusing on the population’s position towards vaccines as well as the socioeconomic factors such as access to vaccines for Māori and Pacific peoples. This study’s goal is to discuss various elements that can influence an anti-epidemic strategy, how they can evolve over time, and what can be inferred from them for the sake of future preparedness, rather than to provide a fly-on-the-wall account of the New Zealand government’s decision-making process.

## 5. Conclusions

The analysis of the anti-COVID-19 strategies implemented by the New Zealand government highlights the diversity of factors that play into decision-making. Epidemiological, geographical, international, social, economic, and governmental factors, as well as the state of the HC system and of potential treatments and vaccines, all impact the choice of a strategy. Independently of the effectiveness of an initial choice, the situation is bound to evolve and strategies must be re-evaluated and adapted accordingly. Therefore, what was once an elimination strategy praised worldwide can later become ineffective. In New Zealand, a solution as strict as elimination did not prove itself to be longer sustainable in the context of the COVID-19 pandemic despite being particularly effective for 16 months. A switch to a strategy similar to suppression with less stringent measures was required to better address the long-term evolution of the situation. Lessons from what worked, and what did not, can however inform the future creation of plans to prepare for pandemics.

## Data Availability

Publicly available datasets were analyzed in this study. This data can be found here: [https://github.com/owid/covid-19-data/tree/master/public/data] (accessed on 11 January 2022).
